# P-796. The Deadly Raise of NTM : An Observational Study on Clinical profile and Outcome Of NTM Disease

**DOI:** 10.1093/ofid/ofae631.988

**Published:** 2025-01-29

**Authors:** Vasireddy Teja, Bibhuti Saha, Soumendranath Haldar

**Affiliations:** school of tropical medicine, Kolkata, West Bengal, India; school of tropical medicine, Kolkata, West Bengal, India; school of tropical medicine, Kolkata, West Bengal, India

## Abstract

**Background:**

NTM is ubiquitously present in the environment. When these microorganisms shed, they produce biofilms that increase the likelihood of infection.

CLINICAL AND RADIOLOGICAL FEATURES OF NTM

**Figure:**
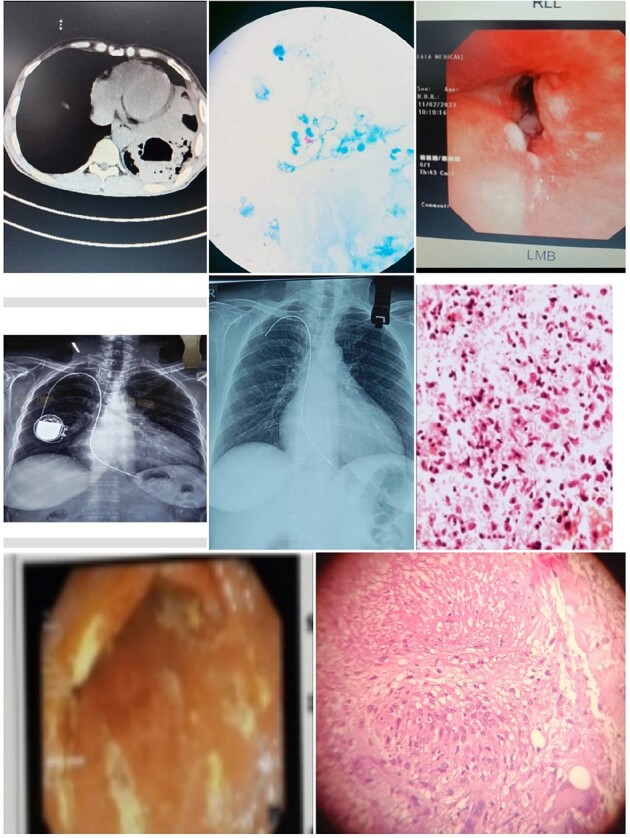

**Methods:**

STUDY PERIOD: 1 YEAR

STUDY DESIGN:

Cross-sectional observational study

STATISTICAL ANALYSIS:

Categorical variables will be expressed as %

NTM VARIOUS CLINICAL SITES

**Figure:**
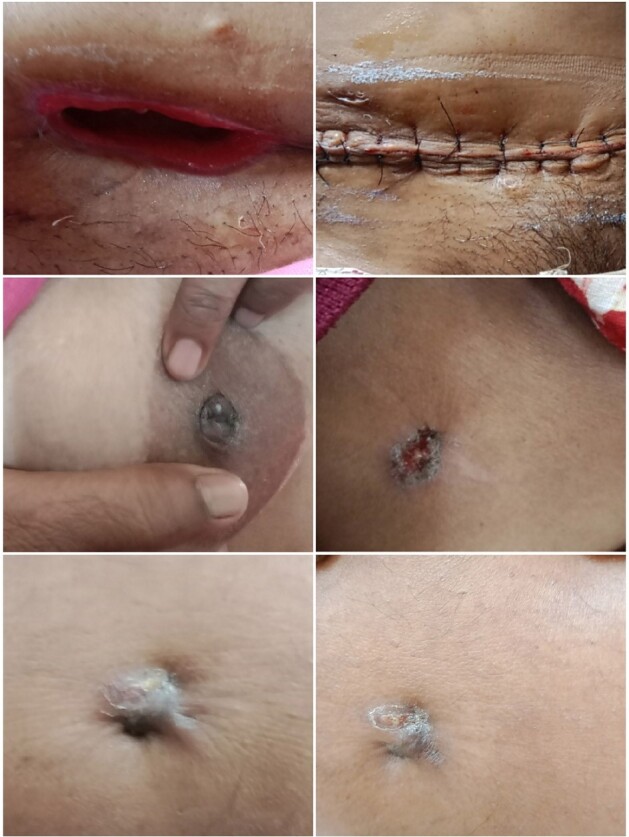

**Results:**

25 cases were selected for analysis. Out of 25 (40% ) were port site infection and 12 % pulmonary. Most common symptom was discharge (60%) . Empirical antibiotics were started in 100% . ID referral was sent in all .RF were seen in 40%. Scans was showing sinus tract formation or deep seated abscess among (80%); . AFB stain was positive in 80% cases and CB-NAAT negative in 100% cases. Culture positivity in 56% , among 15 samples sent for PCR it was positive in 10 (66.6%); among 10 cases sent for sequencing 8(80%) cases were positive. Rapid growers 92% and then slow growers were seen in 8% cases. most common species was M.chelonae (28.5%) followed by M.fortuitum and abscessus (21.4%); among 3 species of abscessus sub species were able to identify in one case and it was boleti 33.3%. slow growers MAC and kansasi were identified 7% each. NTM culture was given positive but species identification was not done in 14.2% cases. Culture was negative in 44% of cases. DST was done in 14 cases and genotypic was able to do in 5 cases and *erm* gene detected (40%) cases. Phenotypic DST was done in all culture positive isolates(n=14), among them cotrimoxazole (92.8%); Amikacin and linezolid was sensitive in 85.7% isolates, imipenem,levofloxacin and tigecycline in 71.4% and macrolide was sensitive in 57.1% cases only. Various combination regimens were used and most common combination used were amikacin,levofloxacin,macrolide and amikacin,linezolid,levofloxacin and amikacin,levofloxacin,cotrimoxazole in 24% cases; and 1 case had treatment with amikacin,linezolid,tigecycline . 40 % cases used combination treatment for 1 year or longer;20 % cases only used 6 months of therapy. Side effects like GIT intolerance in 80% cases; renal toxicity was seen in 40% cases. Upon follow up death in 8%; resolution in 76% , culture negativity 71.4% non-resolving/recurrence 16%

**Figure:**
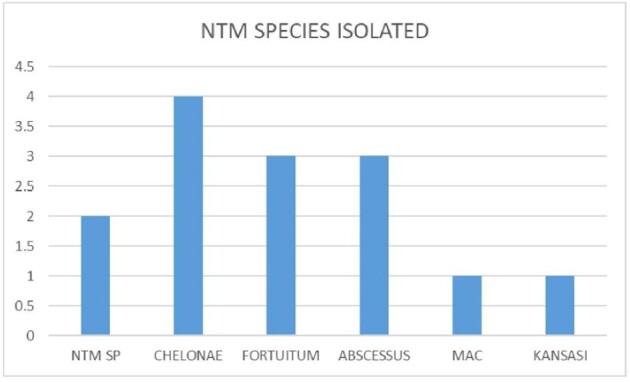

**Conclusion:**

Most of the patients with NTM are misdiagnosed and are treated as tuberculosis

ANTIBIOTIC SUSCEPTIBILITIES AMONG VARIOUS NTM ISOLATED

**Figure:**
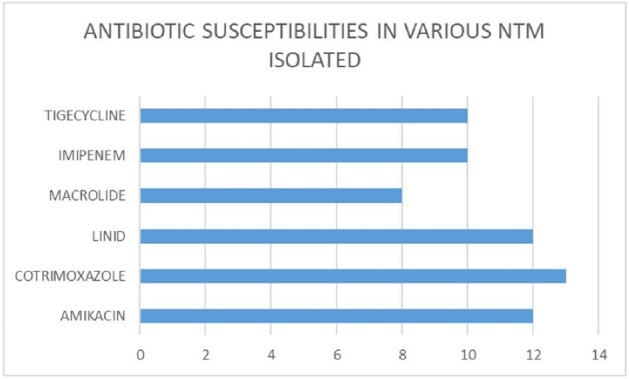

NTM IMAGE 4

**Disclosures:**

**All Authors**: No reported disclosures

